# Tight Junction-Related Barrier Contributes to the Electrophysiological Asymmetry across Vocal Fold Epithelium

**DOI:** 10.1371/journal.pone.0034017

**Published:** 2012-03-19

**Authors:** Qianru Zhang, Kimberly Fisher

**Affiliations:** 1 Experimental Center of Functional Subjects, College of Basic Science, China Medical University, Shenyang, Liaoning, China; 2 Department of Communication Sciences and Disorders, Northwestern University, Evanston, Illinois, United States of America; Biological Research Centre of the Hungarian Academy of Sciences, Hungary

## Abstract

Electrophysiological homeostasis is indispensable to vocal fold hydration. We investigate tight junction (TJ)-associated components, occludin and ZO-1, and permeability with or without the challenge of a permeability-augmenting agent, histamine. Freshly excised ovine larynges are obtained from a local abattoir. TJ markers are explored via reverse transcriptase polymerase chain reaction (RT-PCR). Paracellular permeabilities are measured in an Ussing system. The gene expression of both TJ markers is detected in native ovine vocal fold epithelium. Luminal histamine treatment significantly decreases transepithelial resistance (TER) (N = 72, p<0.01) and increases penetration of protein tracer (N = 35, p<0.001), respectively, in a time-, and dose-dependent fashion. The present study demonstrates that histamine compromises TJ-related paracellular barrier across vocal fold epithelium. The detection of TJ markers indicates the existence of typical TJ components in non-keratinized, stratified vocal fold epithelium. The responsiveness of paracellular permeabilities to histamine would highlight the functional significance of this TJ-equivalent system to the electrophysiological homeostasis, which, in turn, regulates the vocal fold superficial hydration.

## Introduction

Vocal quality, the effort required for producing vocal sound, and laryngeal defense against inhaled particulates are directly correlated with the hydration of the vocal fold [1], [2], [3], but the knowledge of the regulation of vocal fold hydration remains incomplete. Wet stratified squamous epithelium of the vocal folds has been shown to generate a lumen negative potential difference indicating active transepithelial ion and solute fluxes [4]. Water fluxes coupled to transepithelial ion movements and driven by osmotic gradient contribute to vocal fold superficial liquid. Thus, the electrophysiological property of the vocal fold epithelium is one potential regulatory mechanism for the surface hydration of vocal fold. The homeostasis of the bioelectrical gradient in vocal fold epithelium depends on active vectorial ion transport through transcellular pathways, and may also depend on a possible diffusion barrier in paracellular pathways [5], [6]. The focus of the present study is to examine whether TJ-related barrier function is involved in the maintenance of bioelectrical asymmetry in vocal fold.

The molecular elements of the TJ include occludin, claudins and junction adhesion molecules (JAMs) [7], [8], [9]. In addition to these integral proteins, ZOs, cingulin, 7H6 antigen, Rab3b, and symplekin are peripheral proteins forming the cytoplasmic plaques [10], [11], [12], [13], [14]. Among the multiple components of TJ, occludin and ZO-1 are reliable structural and functional markers. Occludin is a universal component of the TJ in most types of epithelia without tissue and species specificity, and there is no direct evidence that occludin exists outside the TJ strands [15], [16]. Also the amino acid sequences of occludin across three mammalian models (human, murine, and canine) are closely related to each other (∼90% identity), a rather high conservation level suggesting its functional significance [17]. Compared to occludin, peripheral protein ZO-1 is less specific for the TJ, since it may also be associated with the adherens junction (AJ) [8], [16]. Nevertheless, ZO-1 belongs to the membrane-associated guanylate kinase homologs (MAGuKs) that bear multiple protein-binding domains. It also has a unique proline-rich domain toward the carboxyl-terminal [18]. Fanning et al. found that the unique proline-rich region of ZO-1 cosediments with a subset of F-actin filaments that terminate at the cell-cell contacts. Also, ZO-2 and the carboxyl-terminal peptide (150 aa of its cytoplasmic tail) of occludin bind to the amino-terminal half of ZO-1 [19]. Thus, ZO-1 acts as a cross-linker between occludin and the actin-based cytoskeleton suggesting that it organizes both structural and signaling components of the TJ. Also, the local co-distribution of occludin and the TJ plaque protein ZO-1 in certain keratinocyte colonies is coincident with TJ morphology on EM [15]. The gene expression level and organization of occludin and ZO-1 are critical determinants of TJ related barrier function.

Histamine, a type of inflammatory mediator, is involved in the pathophysiology of infection, diabetes and allergic diseases, resulting in increased paracellular permeability and edema formation [20], [21]. As the components of TJ have been better defined, the mechanisms whereby histamine compromises TJ-related barrier function are further investigated on a molecular basis. Data on cultured human nasal epithelial cells indicated that 4 hours of 10^−4^M histamine treatment would reduce the gene expression of ZO-1 by ∼50%. This suggested that histamine released in the early stage of nasal hypersensitivity may increase the paracellular permeability of the mucosa by reducing ZO-1 mRNA [22]. Thus, TJ-associated proteins are highly regulated and response to inflammatory mediators. There have been few direct studies of histamine on stratified vocal fold epithelium in the maintenance of bioelectrical asymmetry. Studies concerning the effects of pathogens or inflammatory mediators on TJ-related barrier function will identify new pathogenic mechanisms and potential treatment alternatives in clinical practice.

In the present study, we investigate the effects histamine on the TJ-related barrier and the expression of TJ markers in the vocal fold epithelium. To investigate TJ-related barrier function, transepithelial electrical resistance (TER) and permeability serve as reliable tools to study its ion and size selectivity in vocal fold epithelium. Variations in TER are usually attributed to changes in the permeability of the paracellular pathways [23]. Measurements of the permeability allow us to determine the size selectivity of the TJ-related barrier function to non-charged molecules. Due to relatively larger pores and low resistance in the paracellular pathways, it is easier for non-charged tracers with different molecular weights to cross the epithelia via paracellular pathways. Consistent with the TJ-related barrier function, interference with TJ integrity by external stimuli, such as histamine, would result in certain pathophysiological conditions in native vocal fold epithelium. Possible consequences include increasing the back-diffusion of solutes and water across transporting epithelia which, in turn, reduces the electrical and osmotic gradients that drive absorption and/or secretion across vocal fold epithelium. Histamine-induced increase of vocal fold permeability may facilitate the contact of luminal pathogens and subepithelial antigen-presenting cells. Still in the literature little is known about TJ-related pathophysiological regulation mechanism secondary to extracellular stimuli. Also, experimental manipulation of TJ-related barrier function may also provide an alternative route to enhance the delivery of therapeutic compounds in the airway surface liquid (ASL). Our understanding in these areas would certainly grow with the clarification of regulatory mechanisms of TJ-related barrier function in the paracellular pathways.

## Methods

### Tissue acquisition and preparation

Fresh ovine larynges and kidneys are obtained from a local abattoir (Chiappetti's slaughterhouse, Chicago, IL) and immediately immersed in 4°C Hanks' balanced salt solution (HBSS) for transport to our laboratory. Vocal fold epithelia and renal pyramids are dissected, washed with 0.1% diethyl pyrocarbonate (DEPC) treated distilled water, weighed, recorded, labeled, snap-frozen in liquid nitrogen, and stored at −80°C for total RNA isolation.

### RT-PCR

Total RNA is isolated from ovine vocal fold epithelium and renal pyramids using RNA stat-60 reagent (Tel-Test Inc., Friendswood, TX). Primers for occludin, ZO-1, and GAPDH are listed in Table 1. Then first strand cDNAs are synthesized and RT-PCR is carried out. The 50 µl PCR reaction system contains 25 µl 2XPCR ReadyMix, 3 µl 25 mM MgCl_2_, 0.5 µl sense primer, 0.5 µl antisense primer, 2 µl RNA/cDNA template, and 19 µl sterile water. All PCR conditions are as follows: 30 cycles of denaturation (60 seconds), annealing (45 seconds), and extension (60 seconds), followed by a final extension (5 minutes). An aliquot from each PCR amplified product is electrophoresed on 1.5% agarose gel, stained with 0.1 µg/ml ethidium bromide (EtBr), examined and photographed in a 302 nm UV transilluminator (Kodak electrophoresis documentation and analysis system, Carestream Health, Inc., New Haven, CT 06511).

**Table 1:** Sequence of primers for occluding, ZO-1, and GAPDH.

Primer	Sequence	Product(bp)
occludinF	5′-GATCAGGGAATATCCACC-3′	199
occludinR	5′-ATTGTACTCGTCAGCAGC-3′	
ZO-1F	5′-AGAAGATAGCCCTGCAGC-3′	252
ZO-1R	5′-AGTCCATAGGGAGATTCC-3′	
GAPDHF	5′-TTGTCAGCAATGCCTCCTGC-3′	430
GAPDHR	5′-TCGCTGTTGAAGTCGCAGG-3′	

### TER

We obtain electrophysiological measures using a commercially available Ussing chamber (Model 15362, World Precision Instruments, Sarasota, FL, USA) [4]. Forty-six vocal fold mucosae are randomly assigned to the experimental group and sham control group. For the experimental groups, immediately after the baseline measurements are obtained, the luminal side of the chamber is filled with histamine (catalog#H7125, Sigma-Aldrich Inc., Saint Louis, MS) with a final concentration of 10 µM, 1 mM, and 0.1M, respectively. For the sham control group, HBSS is added instead of histamine. The process of the addition takes less than 1 minute and the corresponding TER is recorded at 1 and 2 hours after exposure to histamine or control reagent as described above. The pH value of HBSS bathing buffer is 7.3±0.21 (n = 9) at room temperature. Histamine addition up to 100 mM doesn't significantly change the pH value of HBSS buffer (7.2±0.22, n = 9, p = 0.167>0.05).

### Permeability to horseradish peroxidase (HRP)

Thirty-six vocal fold mucosae are randomly assigned to the experimental group and sham control group. For the experimental group, immediately after the baseline electrophysiological measurements are obtained, the luminal side of the chamber is filled with histamine with a final concentration of 1 mM and 0.1M, respectively, together with HRP (Signa-Aldrich Inc., Saint Louis, MS) given a final concentration of 7.6unit/ml. For the sham control group, HBSS is added instead of histamine solution together with HRP. Immediately after the addition of HRP and at 1 and 2 hours after exposure to histamine or sham control reagent, 100 µl bathing solution from the luminal and basal side of the Ussing chamber is collected for the spectrophotometrical examination. The concentration-response data at 410 nm gave a standard curve at 5 second of the reaction time as Abs = 0.324×Lg[HRP]+0.938 (r = 0.98, p<0.001). For each sample, the measurement is performed in triplicate manner simultaneously and the resultant mean absorbance is recorded as the final result. The corresponding TER is recorded in order to verify integrity and viability of the vocal fold mucosa.

### Statistics

A series of full factorial repeated measure two way analyses of variance (ANOVAs) are used (SPSS for Windows, 10.0.1, standard version, SPSS Inc.). And Tukey HSD and Bonferroni post hoc tests are performed to obtain pairwise post hoc comparisons. For all tests, p<0.05 is considered statistically significant. Data are shown with standard deviation of the mean.

## Results

### RT-PCR

**Figure 1:**
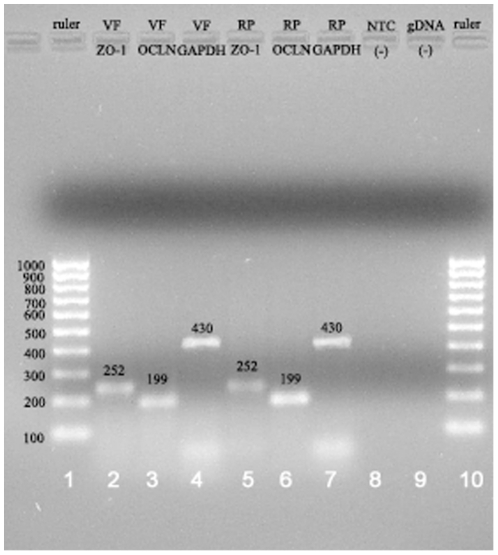
Electrophoresis results of occludin, ZO-1, and GAPDH mRNA in the native ovine vocal fold epithelium and ovine renal pyramids. Equal volumes (4 µl) of RT-PCR products from each sample were separated by electrophoresis on a 1.5% agarose gel and stained with ethidium bromide. Lane 1 and 10 show EZ load 100 bp molecular ladder (5 µl) increasing in size by 100 bp. Single PCR product of 252 bp with ZO-1-specific primers is detected in native vocal fold epithelium and renal pyramid (lane 2 and 5). Single PCR product of 199 bp with occludin-specific primers is also observed in both tissue groups (lane 3 and 6). GAPDH-specific primers extract PCR products of 430 bp with similar intensity from both tissue samples (lane 4 and 7). NTC and gDNA contamination controls do not bear any visible dsDNA band (lane 8 and 9).

RT-PCR analysis (Figure 1) reveals gene expression of TJ-associated proteins, occludin and ZO-1, in native ovine vocal fold epithelium.

### TER

**Figure 2:**
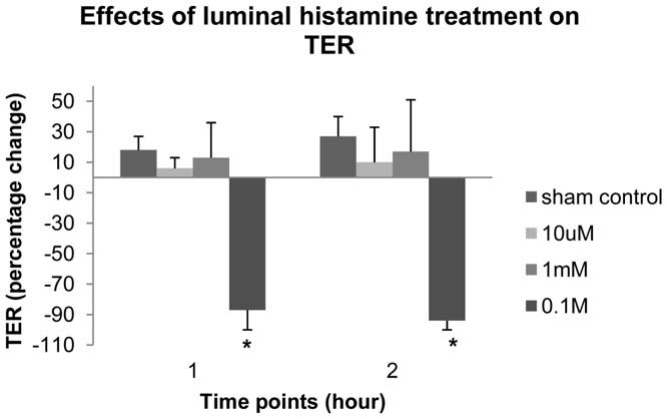
Luminal histamine treatments result in decreased TER. 0.1M luminal histamine treatment significantly (p<0.05) decrease TER of native ovine vocal fold epithelia. Data represent the mean and standard deviation of percentage change in TER. The baseline value for TER (time 0) was 410±173.9 Ω·cm^2^.

The electrophysiological data show that luminal histamine treatment compromises TJ-related barrier function to charged solutes in a time- and dose-dependent manner (Figure 2). Under the control condition (n = 14), TER increases about 27% in the first 2 hours after its baseline value is reached. Luminal histamine treatments of 10 µM (n = 9) and 1 mM (n = 12) slow down this increasing TER to about 10% and 17% after 2 hours of treatments. TER decreases significantly after luminal application of 0.1M histamine to the vocal fold mucosae in a time-dependent manner compared to that of the control condition (ANOVA, tests of between-subjects effects, main effect, treatment, [F(1, 3) = 116.723, p<0.000]; ANOVA, tests of within-subjects contrasts, interaction effect, time points×treatment conditions, [F(1, 3) = 91.938, p<0.000]). Luminal histamine treatment (0.1M) significantly decreases the TER by 94% of its baseline value in a time-dependent manner (Tukey HSD & Bonferroni post hoc, p<0.000).

**Figure 3:**
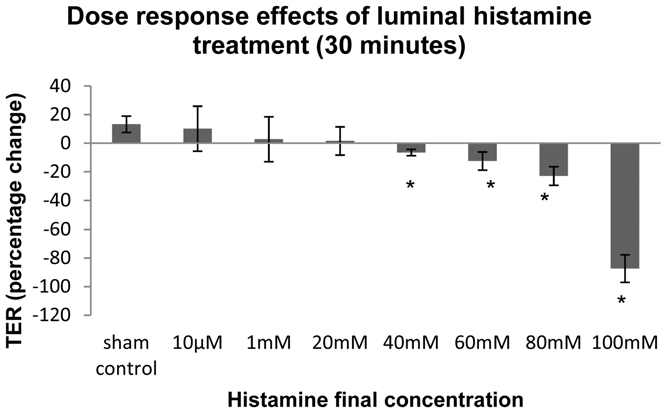
Dose-response effects for 30 minutes luminal histamine treatment. Histamine (40 mM to 100 mM) significantly (p<0.05) decreases TER in native ovine vocal fold epithelium in a dose-dependent manner. Data represent the mean and standard deviation of percentage change (%) in TER.

We notice that the two lower doses of histamine only slowed down the increase of TER, while the high dose of histamine would significantly decrease TER by 94%. To investigate whether histamine-induced reduction in TER in ovine vocal fold epithelium is dose-dependent, we did a series of concentration-response experiments using different histamine doses ranging from 1 mM to 100 mM with 20 mM increment. Data (Figure 3) show that thirty minutes of luminal histamine treatment [40 mM(−6.47%±2.21%), 60 mM(−12.44%±6.32%), 80 mM(−22.92%±6.52%), and 100mM(−87.32±9.66%)] would significantly decrease TER in a dose-dependent manner (P<0.00 for the four doses). Histamine exhibits a maximal effect between 20 mM and 100 mM, and concentrations above this have no greater effect. At a concentration of 20 mM or less, TER does not significantly differ from that of sham controls in ovine vocal fold epithelium. We also performed A-B-A withdrawal single-case experimental designs with the two upper histamine doses, 60 mM and 80 mM (unpublished data). Histamine at these doses would reversibly reduce TER without sacrificing the bioelectrical viability of the vocal fold epithelium, arguing in favor of increased ion permeability of TJ-related barrier function.

### Permeability to HRP

**Figure 4:**
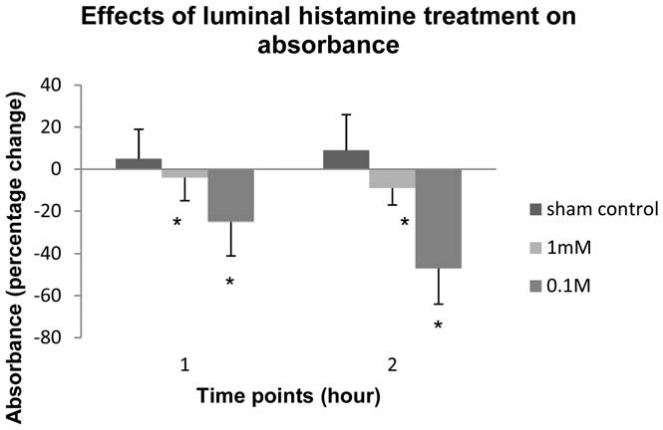
Luminal histamine treatments increase penetration of HRP in ovine vocal fold epithelium. Luminal histamine treatments significantly (P<0.05) decrease absorbance of samples from the luminal chamber of Ussing system in a dose- and time-dependent manner. Data represent the mean and standard deviation of percentage change in absorbance. The baseline absorbance is 0.3604±0.1555 at 410 nm.

Absorbance of samples from the luminal chamber, consistent with HRP concentration, significantly decreases after luminal application of histamine to the vocal fold mucosae in a time- and dose-depend manner compared to that of the control condition (ANOVA, tests of between-subjects effects, main effect, treatment, [F(1, 2) = 34.027, p<0.000]; ANOVA, tests of within-subjects contrasts, interaction effect, time points×treatment conditions, [F(1, 2) = 43.72, p<0.000]) (Figure 4). Under the control condition (n = 12), the absorbance in the luminal chamber increases by about 9% after 2 hours because of the evaporation of the solvent. In contrast, 1 mM (n = 12) and 0.1M (n = 11) luminal histamine treatments significantly decreases the absorbance in the luminal chamber by 9% and 47% in a time-dependent manner, respectively (Tukey HSD post hoc, p<0.044 & p<0.000). No absorbance was detected in samples from the basal chamber.

## Discussion

The present study demonstrates the expression of TJ-associated proteins, occludin and ZO-1 in ovine vocal fold epithelium. Also, histamine significantly decreases TER accompanied by increased penetration of HRP, in a time- and dose-dependent fashion. Thus, a TJ-equivalent system [15] with typical TJ-associated composition may exist and contributes to the maintenance of electrophysiological gradients across the vocal fold epithelium. These data argue in favor of roles of functional TJ-related barrier in ovine vocal fold epithelium.

Recent studies highlight the importance of TJ-related barrier function in squamous stratified epithelia [5], [6], [24], [25]. As to histamine's effect on TJ-related barrier function, in 1961 Majno and Palade first suggested that histamine increased the permeability of blood vessels in rat via opening the inter-endothelial-cell junction [26]. TER of pial microvessels in anaesthetized rats was about 1800 Ω·cm^2^ indicating a tight barrier with very low ion permeability. Addition of 10^−4^M and 10^−3^M histamine to the bathing solution caused a rapid and reversible 75% and 81% decrease in TER, respectively, suggesting a leaky barrier incapable of maintaining brain ion homeostasis [27], [28]. Studies on in situ brain microvessels in cats showed that luminal or brain side application of histamine (10^−9^–10^−3^M) caused a relatively nonselective increase in permeability to tracers of up to 150 kDa molecular weight [29]. Here we demonstrate a significant decrease in TER when bathing the luminal surface of excised ovine vocal fold mucosae with 40 mM to 0.1M histamine in a dose-dependent manner. Also, 1 mM and 0.1M luminal histamine treatments increased the permeability to HRP in a time- and dose-dependent manner. The bioelectrical and permeability data in the current study agree well with the results of the preceding studies and indicate that histamine is able to compromise TJ-related paracellular permeabilities in ovine vocal fold epithelium. In other words, TJ-related barrier function is involved in the maintenance of bioelectrical asymmetry in vocal fold epithelium. These data provide provocative evidence for the TJ-related barrier function controlling paracellular ion and solute fluxes in stratified epithelia.

The effective doses of histamine imposing significant compromise on the TJ-related paracellular barrier function are variable depending on epithelial types, techniques, methods, and species. In cultured human umbilical vein endothelial cells, dose-dependent effects of histamine had been manifested in a variety of research paradigms. Histamine (10^−5^ to 10^−3^M) increased the paracellular permeability to Evans blue with a maximum effect of 10^−4^M in cultured endothelial monolayers [30]. Also, histamine (10^−7^ to 10^−3^M) stimulated second messenger, inositol-1,4,5-triphosphate (IP3), accumulation with a maximal effect around 10^−4^ to 10^−3^M [31], [32], [33]. Similar doses of histamine would also take effect in other endothelial cells. Addition of 10^−4^M and 10^−3^M histamine to the bathing solution caused a rapid and reversible 75% and 81% decrease in TER of pial microvessels in anaesthetized rats, respectively [27], [28]. However, results from epithelia were controversial owing to epithelial types and species. To study the effects of chemical mediators on canine tracheal epithelium, researchers collected TER in a similar manner as the current study using Ussing-type chambers. Luminal histamine (10^−4^M) treatment did not alter TER significantly [34]. Also, luminal histamine (10^−4^M to 2×10^−2^M) caused an increase in TER in human bronchial epithelial cultures with a maximal change by 6-hour-incubation [35]. While 10^−4^M histamine would reduce the gene expression of ZO-1 in cultured human nasal epithelial cells [22]. In native ovine vocal fold epithelium, 10^−3^M histamine was able to increase the paracellular penetration of HRP, but the effective doses for significant TER reduction were around 4 to 10×10^−2^M. Therefore, we acknowledged the difference in histamine application concentration when dealing with distinct epithelial models and species.

It is considered that the gene expression of TJ-associated proteins is highly regulated and responded to inflammatory mediators modulating TJ-related barrier function. The correlation between the gene expression of occludin and/or ZO-1 and paracellular permeability had been demonstrated by previous studies. The property of TJ-related paracellular permeability corresponded inversely with the gene expression level of occludin [36], [37]. Similarly, a correlation between ZO-1 protein content and TER was found in rat cultured cerebral endothelial cells and retinal vascular endothelial cells [38], [39]. Further work is necessary to ascertain whether these changes of gene expression were direct effects of histamine on occludin and/or ZO-1 turnover.

Due to abundant knowledge on transepithelial ion and water transportation in the airway [4], [40], [41], [42], [43], [44], sheep is considered as an appropriate mammalian model for the current study. Sheep has a low incidence of airway abnormality. Ovine tissue is more cost-effective compared to canine tissue because it is readily available from slaughterhouses. Most measurements of ovine laryngeal skeleton are within the range of human larynx [45], [46], which makes ovine model rather practical in the lab. Despite the lack of a definite border between the true and false vocal folds, the transmural potential difference of ovine model is on the similar magnitude as human measurement. Using the nucleotide sequence for *Ovis aries* extracted from NCBI GenBank® (the NIH genetic sequence database), we perform sequence similarity analysis for ovine TJ-associated proteins, occludin (gi: 14475566) and ZO-1 (gi: 14475560). By comparing the known ovine partial mRNA sequence to other species, we notice that the nucleotide sequence of occludin is 98% identical to cow, 97% identical to dog, 94% to human, 89% to mouse, and 84% to rat. The result for ovine ZO-1 is 97% identical to cow, 96% identical to human, 93% to dog, and 90% to mouse and rat. Such a nucleotide sequence similarity indicates a high conservation across species for both occludin and ZO-1, which also helps us to justify the choice of sheep.

Pathophysiological and therapeutic implications of the current study are also worthy of consideration. Consistent with the TJ-related barrier function in the paracellular pathways in vocal fold epithelium, interference with TJ integrity by external stimuli, such as histamine, would result in certain pathophysiological conditions. Consequences would include increasing the back-diffusion of solutes and water across transporting epithelia which, in turn, would reduce the electrical and osmotic gradients that drive vocal fold absorption and secretion. Histamine induced increase in vocal fold permeability may also facilitate the contact of luminal pathogens and subepithelial antigen-presenting cells. But still little is known about TJ-related pathophysiological regulation secondary to extracellular stimuli. Experimental manipulation of this barrier function may also enhance the delivery of therapeutic compounds. Our understanding in these areas would certainly grow with the clarification of regulatory mechanisms of TJ-related barrier function in the paracellular pathways. Therefore, one of the properties of vocal fold epithelium may be to allow modulation of permeability in response to local chemical signals. Since the maintenance of local bioelectrical asymmetry is a potential mechanism for superficial hydration of vocal fold, improved understanding of the modulation of paracellular permeability would give insights into the value and endogenous mechanisms of vocal fold epithelium in maintaining a dynamic and responsive barrier. The clarification of these endogenous mechanisms for deliberate manipulation of the paracellular permeability would certainly bear therapeutic significance including facilitation of drug delivery and prevention of antigen sensitization.

In summary, we conclude that TJ-related paracellular permeability exists and contributes the bioelectrical homeostasis of the vocal fold epithelium. Also, applications of inflammatory mediator, histamine, improve the understanding of physiological and pathophysiological mechanisms underlying the surface hydration of the vocal fold.
